# Male adolescents with contralateral blebs undergoing surgery for primary spontaneous pneumothorax may benefit from simultaneous contralateral blebectomies

**DOI:** 10.1186/s12890-021-01577-5

**Published:** 2021-07-03

**Authors:** Chieh-Ni Kao, Shah-Hwa Chou, Ming-Ju Tsai, Po-Chih Chang, Yu-Wei Liu

**Affiliations:** 1 Department of Surgery, Kaohsiung Medical University Hospital, Kaohsiung Medical University, No. 100, Tzyou 1st Road, Kaohsiung, 80756 Taiwan; 2 Department of Surgery, Pingtung Hospital, Ministry of Health and Welfare, Pingtung, Taiwan; 3 Division of Pulmonary and Critical Care Medicine, Department of Internal Medicine, Kaohsiung Medical University Hospital, Kaohsiung Medical University, Kaohsiung, Taiwan; 4 PhD Program in Environmental and Occupational Medicine, College of Medicine, Kaohsiung Medical University, National Health Research Institutes, Kaohsiung, Taiwan

**Keywords:** Primary spontaneous pneumothorax, Video-assisted thoracic surgery (VATS), One-stage VATS, Simultaneous VATS, Contralateral bleb, Adolescent

## Abstract

**Background:**

In adults with primary spontaneous pneumothorax (PSP), contralateral recurrence occurs in about 25–28% when there are asymptomatic blebs. How to treat contralateral recurrence of PSP in pediatric populations remains controversial. This study evaluated the outcomes of excising contralateral blebs to prevent recurrence in adolescents being operated on for PSP under the same anesthesia.

**Methods:**

One hundred thirty-two male PSP patients under age 19 were surgically treated in a single institution between January 2008 and December 2016. Thoracoscopic blebectomies with pleurodesis were performed in all patients. The patients were categorized into those with contralateral blebs receiving one-stage bilateral surgeries (32 patients), those with contralateral blebs only receiving unilateral surgeries (40 patients), and those without contralateral blebs only receiving unilateral surgeries (60 patients). Perioperative details and outcomes were retrospectively analyzed.

**Results:**

Significant differences in contralateral recurrence rate were found among the three groups (0%, 30%, and 1%, respectively; *P* < 0.001). Multivariate analysis showed that being under 16.5 years old was a risk factor for overall recurrence (Hazard ratio [HR] 2.81, 95% confidence interval [CI] 1.08–7.30, *P* = 0.034). Moreover, patients who had contralateral blebs and only received unilateral surgery were at greater risk of overall recurrence (HR 6.06, 95% CI 1.77–20.75, *P* = 0.004). Kaplan–Meier analysis showed that contralateral and overall recurrence-free survival differed among the three groups (*P* < 0.0001, *P* = 0.0002).

**Conclusions:**

Although younger male PSP adolescents treated with surgery were more likely to have postoperative recurrences, the performance of simultaneous contralateral blebectomies in those receiving one-stage bilateral surgeries significantly reduced future contralateral recurrence without compromising patient safety.

## Background

Primary spontaneous pneumothorax (PSP) most often affects healthy young males. While the reported annual incidence of PSP is approximately 21 cases per 100,000 adults [1], that incidence is 3.4 per 100,000 in pediatric populations, peaking between 14 and 17 years of age, mainly in late adolescence [2]. Recurrence has always been a serious problem associated with PSP, yet it remains unclear what factors predispose its recurrence in adolescents and how it can best be managed surgically. There are probably multiple factors that lead to the recurrence in PSP. One widely-accepted risk factor is the rupture of pulmonary blebs or bullae, so the presence of these air-containing lesions on high-resolution computed tomography (HRCT) is thought to predict recurrence and has been investigated extensively [3–5].

Currently, the management of PSP in children is largely based on experience treating adults. A few studies using early video-assisted thoracic surgery (VATS) bullectomy and mechanical pleurodesis for pediatric PSP have reported children undergoing this surgery have less ipsilateral recurrence than those who do not [6–8]. However, it is not clear how to best manage contralateral recurrence in pediatric patients. Ciriaco et al. recommended that VATS be reserved only for the affected side [8]. In CT scans performed by Soccorso et al., 20% (10/49) of their pediatric patients with PSP had asymptomatic contralateral blebs/bullae. Among those with the blebs, 40% developed pneumothorax within six months [9]. Some studies of adult PSP have associated asymptomatic contralateral blebs with a 25–28% risk of future spontaneous pneumothorax [3, 10–12].

Surgery seems to be the most effective method of preventing recurrence. Previously, we reported that our excisions of contralateral blebs significantly lowered contralateral recurrence in adult patients receiving operations for ipsilateral PSP [13]. We were not sure whether the performance of prophylactic blebectomies in pediatric patients with asymptomatic contralateral blebs would have similar benefits. Therefore, we performed a retrospective cohort study to analyze the relevant risk factors for ipsilateral, contralateral, and overall recurrences and outcomes in male adolescent patients surgically treated for PSP during a nine-year period at our medical center. We divided our patient population into those with contralateral blebs receiving one-stage bilateral surgeries (B+cb), those with contralateral blebs only receiving unilateral surgery (U+cb), and those without contralateral blebs only receiving unilateral surgery (U−cb). The primary outcomes of this study were recurrence rates (contralateral, ipsilateral, and overall). Recurrence-free probability and risk factors for the recurrence of pneumothorax were also analyzed.

## Methods

### Study design

**Fig. 1:**
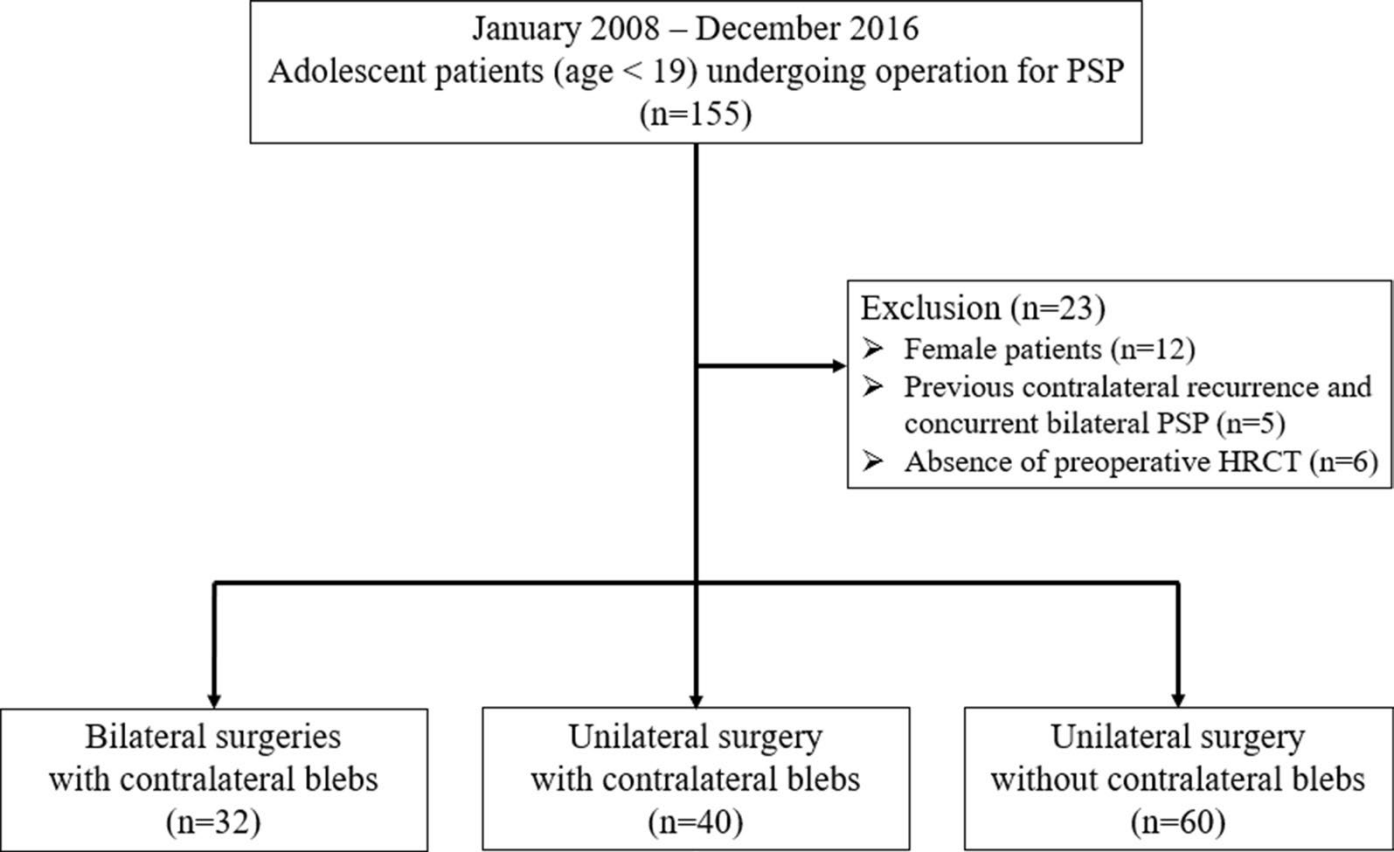
Flow diagram for patient recruitment. *PSP* primary spontaneous pneumothorax, *HRCT* high-resolution computed tomography

This retrospective cohort study was conducted from January 2008 to December 2016 at a single medical center. The Institutional Review Board of Kaohsiung Medical University Hospital approved the study and waived the requirement for written informed consent from all patients (KMUHIRB-E(I)-20190158). We included data for 155 patients aged under 19 years who had received surgeries for PSP. We excluded female patients (n = 12), patients with contralateral recurrence following previous PSP and concurrent bilateral PSP (n = 5), and patients without preoperative chest HRCT results on file (n = 6). Female patients were excluded to rule out the possibility of catamenial pneumothorax and to obtain a homogenous analysis, since the population most affected by PSP are male adolescents. After exclusion, patients were classified into three groups–patients with contralateral blebs receiving one-stage bilateral surgeries (B+cb), patients with contralateral blebs only receiving unilateral surgery (U+cb), and patients without contralateral blebs only receiving unilateral surgery (U−cb) (Fig. 1).

Based on professional guidelines and scientific studies on the management of adult PSP [10, 14–19], surgery was indicated in cases of recurrent ipsilateral PSP, persistent air leaks (> 3 days) after chest tube drainage, and PSP at the first attack exhibiting ipsilateral and/or contralateral blebs on HRCT. We collected patient characteristic data including age, height, weight, body mass index (BMI), smoking habit, perioperative data, and length of hospital stay as well as postoperative recurrence. HRCTs were conducted while the patients were hospitalized when the affected lung was expanded, and were interpreted by a radiologist and thoracic surgeon together to determine whether or not the patients had blebs/bullae. As in our previous study [4, 13], if HRCTs indicated that a patient had contralateral blebs, we fully explained his ailment to him and his guardians. In these cases, we usually bring up the possibility of performing contralateral VATS while carrying out the unilateral PSP operation without trying to persuade the guardians one way or another. Rejection or acceptance of the contralateral preventive surgery is decided solely by the patients and their guardians.

### Operative procedure and postoperative follow-up

All VATS blebectomies or bullectomies with mechanical pleurodesis were performed by board-certified thoracic surgeons using a 2-port or uniportal method as previously described [13]. The operations were performed in the lateral decubitus position under general anesthesia using double-lumen endotracheal tubes for lung isolation. The blebs/bullae were identified carefully over the entire lung surface followed by resection via endostapler. Mechanical pleural abrasion was performed under direct view of the thoracoscopy using curved ring forceps wrapped with a piece of Marlex mesh. For patients without obvious ipsilateral blebs/bullae on preoperative HRCT, we empirically performed apical wedge resection followed by pleurodesis if no air leaks were detected intraoperatively. The contralateral lung was operated on in a similar fashion at the same time one-stage bilateral VATS was scheduled. We routinely use chest tubes (24–28 Fr) and no regular suction after surgery. Chest tube removal is based on evidence of clear pleural drainage and absence of any air leakage.

All patients were advised to seek help in the event of any discomfort or suspicious signs of recurrence. Patients were regularly followed up in our outpatient clinic at 3-month intervals the first two years following surgery. Chest x-rays or HRCT were performed to determine diagnosis of recurrent pneumothorax if the aforementioned conditions developed at any time during follow-up. The patients were also followed up via electronic medical records and telephone interviews in May 2020 to determine the cumulative incidence of recurrence. At this time, all of the patients were contacted and reported any of these conditions related to the recurrence of PSP.

### Statistical analysis

Descriptive variables were expressed as numbers with percentages and continuous variables expressed as means with standard deviations or medians with interquartile ranges (IQR). Continuous variables were compared using analysis of variance (ANOVA) or Kruskal–Wallis test and descriptive variables were analyzed using the Chi-square test. Bonferroni correction was performed to adjust for the effects of multiple comparisons when above-mentioned tests showed a statistical difference between groups. We did not perform propensity score matching (PSM) for the following reasons. The confounding variables in our study were mainly age, height, weight, and smoking, which tended to be homogenous in our patient population of male adolescents. In addition, the perioperative variables included operative time, intraoperative blood loss, and wound pain score inherently differed between groups, since group B+cb (simultaneous bilateral surgeries) had obviously higher values regarding the aforementioned variables.

Multivariable analyses made use of backward elimination. Only variables with a *p* value less than 0.10 were used in the final model. In addition, Cox proportional hazard model was used to identify multivariable risk factors for different recurrence patterns. Cumulative incidence of recurrence and follow-up times were calculated from the date of surgery to the first event of recurrence and analyzed by the Kaplan–Meier method. Log-rank test was used to examine the differences between treatment groups. All statistical operations were performed using the SAS® 9.4 software (SAS Institute Inc., Cary, NC, USA) for Windows. A *p* value less than 0.05 was considered significant.

## Results

**Fig. 2:**
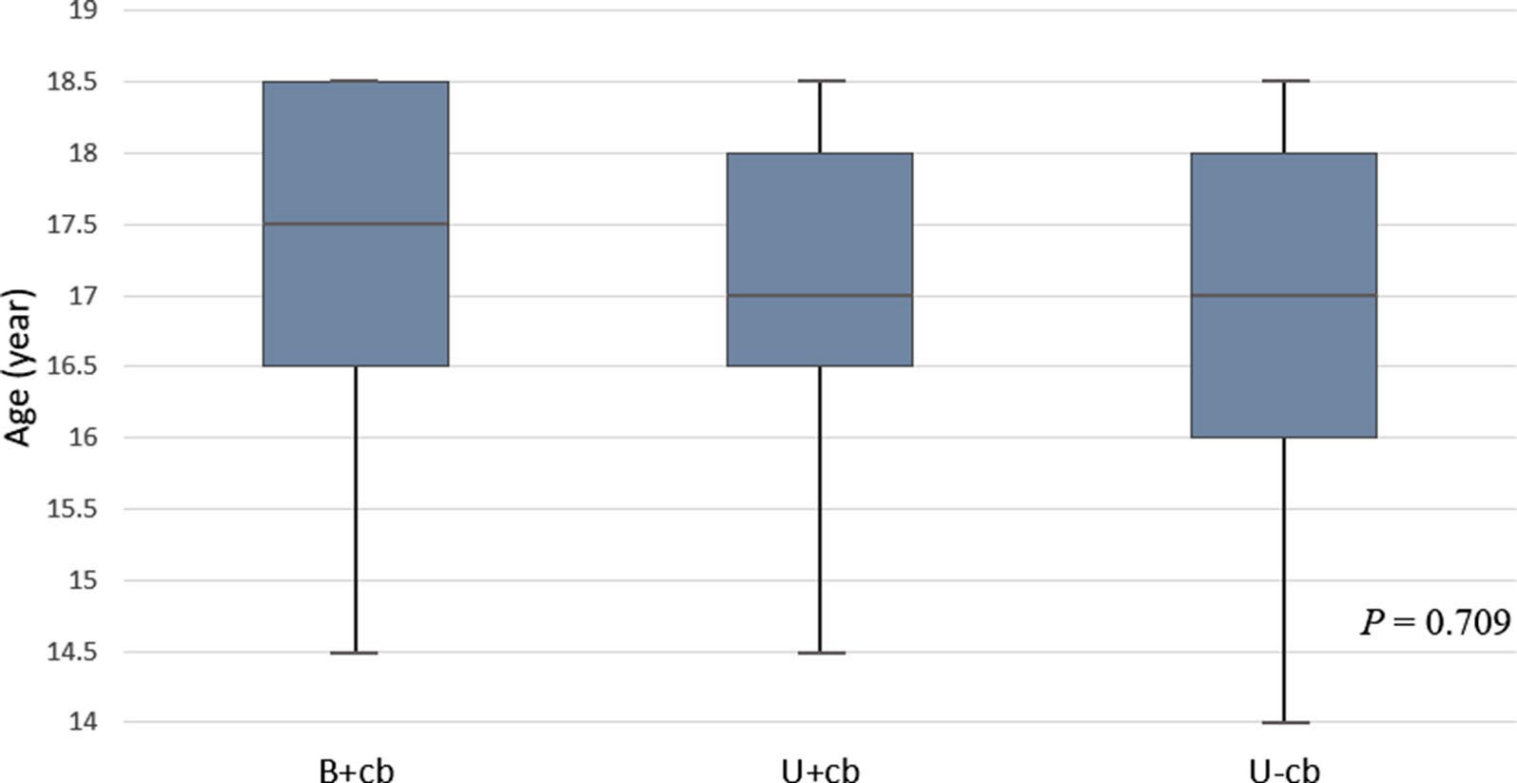
Age distribution of the three groups stratified by 0.5 using box plot analysis. *B*+*cb* those with contralateral blebs receiving one-stage bilateral surgeries, *U*+*cb* those with contralateral blebs only receiving unilateral surgery, *U*−*cb* those without contralateral blebs only receiving unilateral surgery

In total, we enrolled 132 male adolescents with PSP who had received VATS blebectomies with mechanical pleurodesis, had complete preoperative HRCT examinations on file, and were followed up postoperatively for 9 years (2008–2016). Thirty-two patients belonged to group B+cb, 40 to group U+cb, and 60 to group U−cb. There were no significant differences in age, height, weight, BMI, or smoking among the three groups (Table 1). Figure 2 shows the age distribution of the three groups stratified by 0.5 using box plot analysis. Group B+cb had significantly longer operative times and higher postoperative numerical rating scale (NRS) pain scores (both *P* < 0.001) (Table 1). However, there was no significant difference in length of hospital stay and complications among the three groups (Table 1). Based on Clavien-Dindo classification, the most frequent complication was persistent air leakage (persisting for > 7 days postoperatively), followed by pneumonia and wound infection. There was no need to re-operate for any of these complications (grade I and II). The percentage of contralateral recurrence for groups B+cb, U+cb, and U−cb were 0%, 30%, and 1.6%, respectively (Table 1). Notably, the recurrence rate for group U+cb was significantly higher than that of the other two groups (*P* < 0.001). Nineteen patients in total had ipsilateral recurrences, twelve of these patients had reoperations (thoracoscopic wedge resection of the postoperative bullae neogenesis), two chest tube drainages, and five received no procedures but remained under observation. Thirteen patients had contralateral recurrences, for which they all received VATS blebectomies and pleurodesis. There were significant differences in follow-up periods (*P* = 0.002), concordant with the overall recurrence (*P* < 0.001). Group U+cb had the highest number of recurrences and the shortest follow-up periods.

**Table 1:** Patient characteristics with perioperative data

	B+cb (n = 32)	U+cb (n = 40)	U−cb (n = 60)	*p* value
Age (mean ± SD) (y)	17.4 ± 1.0	17.0 ± 1.0	16.9 ± 1.3	0.190
Height (mean ± SD) (cm)	175.3 ± 5.5	174.4 ± 6.2	174 ± 5.6	0.612
Weight (mean ± SD) (kg)	57.6 ± 9	56.8 ± 7.8	57.3 ± 8	0.917
BMI (mean ± SD) (kg/m^2^)	18.7 ± 2.3	18.6 ± 2.1	18.9 ± 2.1	0.852
Smoking (yes), % (n)	19 (6)	8 (3)	10 (6)	0.296
Operative time (median with IQR) (min)	120 (110–120)	60 (50–65)	55 (50–65)	< 0.001
Blood loss (median with IQR) (ml)	25 (20–27.5)	10 (5–15)	10 (5–15)	0.835
Postoperative NRS pain score (median with IQR)	5.5 (5–6.5)	5 (4–5)	4 (3.5–5)	< 0.001
Postoperative hospital stays (median with IQR) (d)	6 (5–7)	4 (4–6)	5 (4–6)	0.255
Follow-up (median with IQR) (mo)	92 (74–121)	51 (14–109)	80 (55–111)	0.002
Complication (grade I and II), % (n)	13 (4)	15 (6)	13 (8)	0.949
Overall recurrence, % (n)	9 (3)	45 (18)	18.3 (11)	< 0.001
Contralateral recurrence, % (n)	0 (0)	30 (12)	1.6 (1)	< 0.001
Ipsilateral recurrence, % (n)	9 (3)	15 (6)	16.7 (10)	0.632

*SD* standard deviation, *BMI* body mass index, *IQR* interquartile range, *NRS* numerical rating scale, *B*+*cb* those with contralateral blebs receiving one-stage bilateral surgeries, *U*+*cb* those with contralateral blebs only receiving unilateral surgery, *U*−*cb* those without contralateral blebs only receiving unilateral surgery. Data are presented as % (n)

**Fig. 3:**
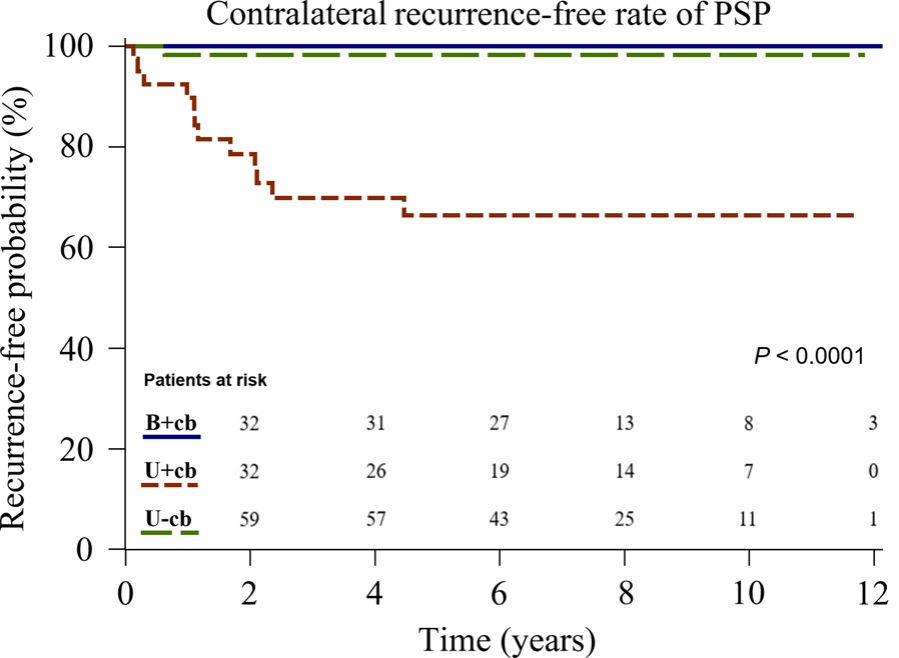
Kaplan–Meier analysis showing contralateral recurrence-free rate of patients with PSP treated in different groups*. PSP* primary spontaneous pneumothorax, *B*+*cb* those with contralateral blebs receiving one-stage bilateral surgeries, *U*+*cb* those with contralateral blebs only receiving unilateral surgery, *U*−*cb* those without contralateral blebs only receiving unilateral surgery

**Fig. 4:**
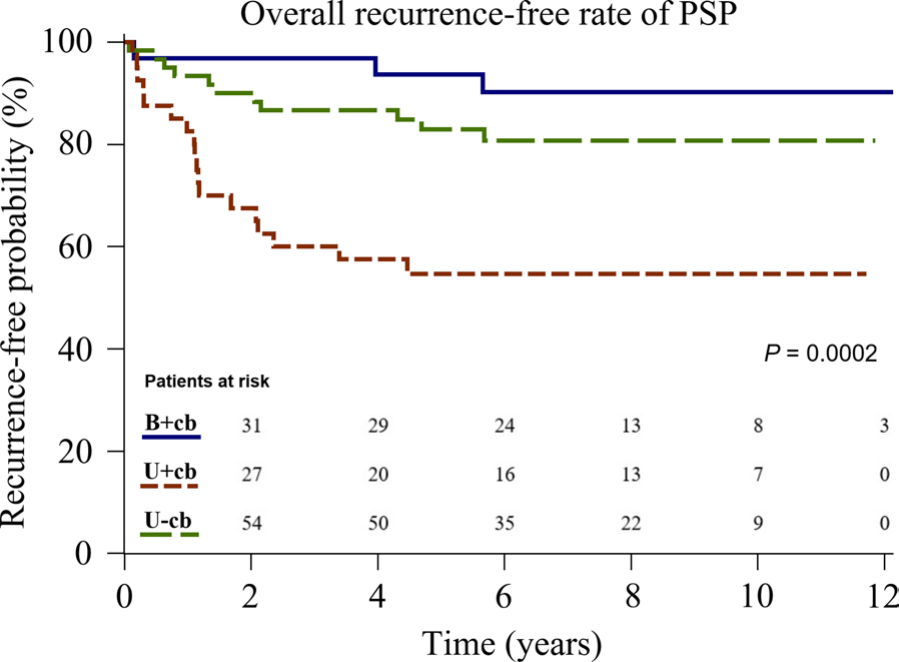
Kaplan–Meier analysis showing overall recurrence-free rate of patients with PSP treated in different groups*. PSP* primary spontaneous pneumothorax, *B*+*cb* those with contralateral blebs receiving one-stage bilateral surgeries, *U*+*cb* those with contralateral blebs only receiving unilateral surgery, *U*−*cb* those without contralateral blebs only receiving unilateral surgery

**Fig. 5:**
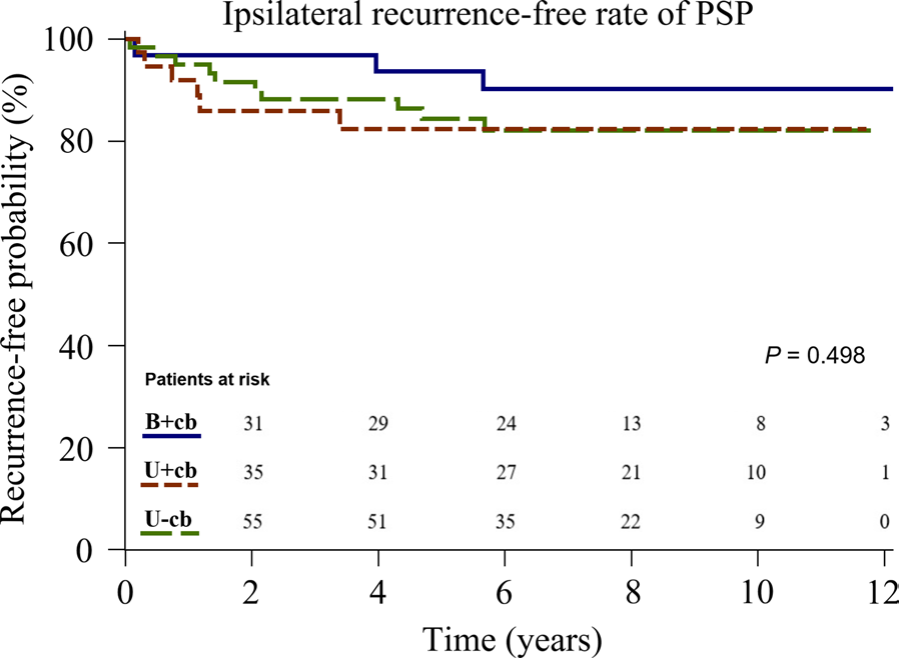
Kaplan–Meier analysis showing ipsilateral recurrence-free rate of patients with PSP treated in different groups*. PSP* primary spontaneous pneumothorax, *B*+*cb* those with contralateral blebs receiving one-stage bilateral surgeries, *U*+*cb* those with contralateral blebs only receiving unilateral surgery, *U*−*cb* those without contralateral blebs only receiving unilateral surgery

As can be seen in Table 2, in both our univariable or multivariable analyses, only age and type of intervention were significant risk factors for overall recurrence. We could not assess risk for ipsilateral and contralateral recurrences because of the small sample sizes. Multivariable analysis revealed the independent risk factors for overall recurrence to be age < 16.5 years (Hazard ratio [HR]: 2.81, 95% confidence interval [CI] 1.08–7.30, *P* = 0.034) and intervention group U+cb (HR 6.06, 95% CI 1.77–20.75, *P* = 0.004). Figures 3 and 4 show the contralateral and overall recurrence-free rate calculated using the Kaplan–Meier method and compared using Log-rank test. Among patients with contralateral blebs, those treated with simultaneous excision of contralateral blebs (group B+cb) had significantly lower contralateral and overall recurrence than those only treated with unilateral surgery (group U+cb) (*P* < 0.0001 and *P* = 0.0002, respectively). Ipsilateral recurrence-free rate was similar among all three groups (*P* = 0.498) (Fig. 5).

**Table 2:** Risk of overall recurrence based on patient characteristics and types of intervention

	Overall recurrence, % (n)	Univariate analysis		Multivariable analysis	
		HR (95% CI)	*p* value	HR (95% CI)	*p* value
Age (y)					
Age < 16.5	34 (11/32)	2.86 [1.11–7.38]	0.030	2.81 [1.08–7.30]	0.034
16.5 ≦ age < 18	27 (14/52)	1.94 [0.78–4.82]	0.151	1.49 [0.60–3.74]	0.393
18 ≦ age < 19	15 (7/48)	Reference		Reference	
BMI (kg/m^2^)					
≥ 18.5	30 (20/67)	Reference		Reference	
< 18.5	18 (12/65)	0.9 [0.45–1.81]	0.777	0.98 [0.49–1.97]	0.950
Smoking					
No	26 (31/117)	Reference		Reference	
Yes	7 (1/15)	0.22 [0.03–1.63]	0.139	0.35 [0.05–2.60]	0.303
Intervention					
B+cb	9 (3/32)	Reference		Reference	
U+cb	45 (18/40)	6.44 [1.89–21.93]	0.003	6.06 [1.77–20.75]	0.004
U−cb	18 (11/60)	2.11 [0.59–7.55]	0.253	1.81 [0.50–6.58]	0.365

*BMI* body mass index, *B*+*cb* those with contralateral blebs receiving one-stage bilateral surgeries, *U*+*cb* those with contralateral blebs only receiving unilateral surgery, *U*−*cb* those without contralateral blebs only receiving unilateral surgery. Data are presented as % (n)

## Discussion

In this retrospective 9-year follow-up study, 72 of 132 patients with unilateral PSP were found by HRCT to have contralateral blebs. The incidence (54.5%) in this study was comparable to incidence rates reported in the literature [3–5]. Studies disagree about predictability of a contralateral episode following a first occurrence of PSP. Three studies have found an association between HRCT detection of blebs/bullae in the contralateral lung after ipsilateral PSP and higher risk of contralateral recurrence, reporting 26%, 26.7%, and 25.8% [3, 10, 11]. According to one recent large retrospective cohort study of 1055 PSP patients reported by Jang et al. [12], the 5-year cumulative incidence of contralateral recurrence reached 28.2% for contralateral asymptomatic blebs/bullae. In that study, the authors suggested that preemptive surgery be considered particularly for patients with multiple blebs/bullae.

We weighed the advantages and disadvantages of unilateral VATS and bilateral VATS in patients with and without contralateral blebs. Group B+cb had longer operative times and greater blood loss due to the need for sequential bilateral procedures performed under the same anesthesia as well as higher postoperative pain scores and longer hospital stays. Despite these differences, there were no significant differences in postoperative complications rates among the three groups. Consequently, we consider it is safe for physiologically fit male adolescents to receive one-stage bilateral operations. The patients and their guardians were satisfied with the outcomes, the most important being prevention of recurrence. In our patients in whom contralateral blebs were detected, group B+cb had significantly lower contralateral recurrence than group U+cb (0% vs. 30%, *P* < 0.001). Our results were comparable to those of the previously-mentioned studies reporting recurrence rates of asymptomatic contralateral blebs/bullae ranging 25–28% [3, 10–12]. To the best of our knowledge, our study is the first to evaluate risk of recurrence and long-term outcomes of simultaneous treatment of contralateral blebs with bilateral VATS for pediatric PSP, especially in a male adolescent population.

Although VATS blebectomy with pleurodesis for pediatric PSP has been found to produce similar treatment outcomes in young adult patients, ipsilateral recurrence seems to be more prevalent in adolescents than in young adults even after surgery [8, 20, 21]. Likewise, the current study found the cumulative incidence of ipsilateral recurrence to be comparable among the three groups (9%, 15%, and 16.7%; respectively). The recurrence rate in this study was, however, much higher than the rate we found in our previous study of young adults (7.1%, 8.1%, and 8.5%; respectively) [13].

Another controversial issue is the correlation between risk factors and pneumothorax recurrence. Factors such as younger age, sex, smoking, prolonged air leakage, low BMI, and HRCT detection of blebs/bullae have been associated with recurrence [22–25]. Cardillo et al. found smoking to be significantly associated with PSP recurrence [26]. Huang et al. found contralateral blebs/bullae and underweightedness (BMI < 18.5) to be predictors of contralateral recurrence [3, 5]. Typically, PSP occurs in tall and thin young males with BMIs indicating underweightedness [27]. In this series, we included only adolescents under 19 years old and excluded 12 female patients, making our findings more relevant to a homogenous population. In addition, we did not find smoking or lower BMI to be significantly associated with risk of recurrence. Only 11.3% (15/132 patients) of our male adolescent patients with PSP were smokers, which can be explained by the lower prevalence of smoking in pediatric population compared to adults [28]. Thus, although smoking might play a role in recurrence, it is not as important in recurrence in adolescent PSP. Furthermore, it is noteworthy that group B+cb had higher smoking habit and lower ipsilateral recurrence rate than group U+cb. This result contradicts the report by Cardillo et al. but is consistent with the findings of by Uramoto and Tsuboshima et al. [29, 30], who reported a lower postoperative recurrence in PSP patients who smoked.

In both our univariate and multivariate analyses, younger age (< 16.5 years) and intervention group U+cb were independent risk factors for overall recurrence (Table 2), a result consistent with the finding of high incidence of recurrence in adolescents in a nationwide population-based study in Taiwan [31]. Physical development has been shown to differ among adolescents. For individuals younger than 16 years, growth rates are higher than they are for 17- or 18-years-olds and growth rates remain steady in individuals over the age of 19 years old [20]. The rapid increase in the vertical dimension of the thorax compared with the horizontal dimension could produce an increase in negative intrathoracic pressure at the apex of lung, which may lead to the formation of subpleural blebs/bullae able to induce PSP if they rupture [32]. This increase may also contribute to higher post-surgery recurrence rates in younger patients. Therefore, some authors suggest that surgery for PSP might be delayed in younger groups (age < 16) [20].

One key strength of our study is its long-term follow-up period (median, 80 months; IQR, 50–113 months). The age distribution among the three groups was also similar (median age 17 years old) (Fig. 2). The result of our Kaplan–Meier analysis revealed that over the half of contralateral recurrences (9/13; 69%) and ipsilateral recurrences (11/19; 58%) tended to occur during the first 2-years after surgery (Figs. 3, 5). Notably, all the overall recurrences occurred within five years, except for one patient in group B+cb and another patient in group U−cb, who had ipsilateral recurrences at 68 and 62 months, respectively (Fig. 4). Hence, we suggest vigilant postoperative follow-ups throughout adolescence because there is a close relationship between this age range and potential physical development and chest dimension growth.

The main limitation of this study is that it is a retrospective study design and patients were not randomized. Therefore, the study has some unavoidable selection bias, including absence of HRCT interpretation for blebs/bullae, patient’s and/or their guardians’ viewpoint toward the preemptive contralateral surgery as well as exclusion of the female PSP patients. Another limitation of this cohort study is that HRCT for measuring reconstruction thickness and interval as well as its interpretation criteria might have changed during the nine-year period. Although surgery for the PSP at our institute always comprises blebectomy and mechanical pleurodesis, how surgeons perform the minimally invasive operative technique could vary.

## Conclusions

Although recurrence is high following surgery for PSP in younger adolescents, the performance of simultaneous contralateral blebectomy in those receiving bilateral surgeries may significantly decrease future contralateral recurrence. Our results suggest that one-stage bilateral VATS can be used for certain patients with contralateral blebs without compromising their safety.

## Data Availability

The datasets used and/or analyzed during the current study are available from the corresponding author on reasonable request.

## Abbreviations

PSP: Primary spontaneous pneumothorax
VATS: Video-assisted thoracic surgery
B+cb: Those with contralateral blebs receiving one-stage bilateral surgeries
U+cb: Those with contralateral blebs only receiving unilateral surgery
U−cb: Those without contralateral blebs only receiving unilateral surgery
HR: Hazard ratio
CI: Confidence interval
HRCT: High-resolution computed tomography
BMI: Body mass index
IQR: Interquartile range
